# Memantine abrogates testicular dysfunction induced by risperidone in rats with a potential role of ERK1/2-Nrf2-caspase-3 signaling pathway

**DOI:** 10.1038/s41598-025-94760-1

**Published:** 2025-04-15

**Authors:** Reham H. Mohyeldin, Ehab E. Sharata, Michael Atef Fawzy, Mina Ezzat Attya, Nermeen N. Welson, Remon Roshdy Rofaeil

**Affiliations:** 1 Department of Pharmacology and Toxicology, Faculty of Pharmacy, Deraya University, Minia, 61111 Egypt; 2 Department of Biochemistry, Faculty of Pharmacy, Minia University, Minia, 61511 Egypt; 3 Department of Pathology, Faculty of Medicine, Minia University, Minia, 61519 Egypt; 4 Department of Forensic Medicine and Clinical Toxicology, Faculty of Medicine, Beni-Suef University, Beni Suef, 62514 Egypt; 5 Department of Medical Pharmacology, Faculty of Medicine, Minia University, Minia, 61511 Egypt

**Keywords:** Antipsychotics, Risperidone, Anti-dementia, Reproductive toxicity, Memantine, Biochemistry, Diseases, Endocrinology, Oncology

## Abstract

Psychosis is usually a substantial global burden with a prevalence of 0.4–2%. On the other hand, 50 million people are suffering from dementia, with dementia-related psychosis affecting approximately 25% of them. The current experiment aimed to investigate the effect of the anti-dementia drug memantine (MEM) on testicular damage and insulin resistance induced by the chronic administration of risperidone (RIS) in rats. Six groups of Wistar albino rats were designated as follows: control, MEM-5 (rats received MEM at 5 mg/kg/day, orally, for 4 weeks), MEM-10 (rats received MEM at 10 mg/kg/day, orally, for 4 weeks), RIS (rats were administered RIS at 2.5 mg/kg/day, orally, for 4 weeks), RIS + MEM-5 (rats received MEM at 5 mg/kg/day, orally, co-administered with RIS as in the RIS group for 4 weeks), and RIS + MEM-10 (rats received MEM at 10 mg/kg/day, orally, co-administered with RIS as in the RIS group for 4 weeks). The duration of the study was 28 days. Serum testosterone, resistin, and adiponectin concentrations were determined. The homeostatic model assessment of insulin resistance (HOMA-IR) was also evaluated. Oxidative stress, inflammatory markers, and immunoblotting of ERK1/2, and Nrf2 were quantified in testicular tissue together with histopathological evaluation and a caspase-3 immunohistochemical study. MEM co-administration increased adiponectin, serum testosterone, GSH, SOD, CAT, and Nrf2 expression while decreasing HOMA-IR, resistin, MDA, NOx, ERK1/2, IL-6, TNF-α, NFĸB, and caspase-3 expression. Furthermore, MEM ameliorated all measured parameters and histopathological changes that occurred in the RIS group in a dose-dependent manner. The primary outcomes were attained by attenuating oxidative stress, inflammation, and apoptosis in the testis caused by chronic RIS administration via regulation of the ERK1/2-Nrf2 signaling pathway. Targeting the ERK1/2-Nrf2 pathway is a potential strategy for addressing testicular injury.

**Supplementary Information:**

The online version contains supplementary material available at 10.1038/s41598-025-94760-1.

## Introduction

Psychosis usually presents as a chronic, severe mental disorder that contributes a substantial burden globally in terms of both morbidity and mortality, with undeniable social and economic impacts attached to its unstoppable increase in prevalence worldwide. It is characterized by a combination of positive, negative, and cognitive symptoms that may affect social interaction, thought processes, and emotional reactions. In recent studies, its prevalence was 0.4–1.3%^1^, around 1.4%^2^, or raised to 2% in other studies^3^.

Risperidone (RIS) is a benzisoxazole derivative that is hepatically metabolized by CYP2D6 to the main active metabolite, 9-hydroxy risperidone. The minimum daily dose is 1 mg, and the average therapeutic dose is 2–4 mg/day^4^. RIS controls positive and negative signs with a good safety profile; however, RIS induces metabolic disorders like insulin resistance^5^ and has also been reported to induce reproductive toxicity^6–8^. RIS caused damage to male reproductivity and significantly decreased the level of testosterone with induction of oxidative stress which was shown by a significant reduction in SOD activity and GSH with an increase in MDA^9^.

Grievously, worldwide, 50 million people are suffering from dementia, with dementia-related psychosis affecting approximately 25% of those who have the disease. Furthermore, Alzheimer’s disease is the most common neurodegenerative dementia^10^.

Memantine (MEM) is a voltage-dependent, noncompetitive N-methyl-D-aspartate (NMDA) receptor antagonist that has been registered for the treatment of moderate-to-severe Alzheimer’s dementia with good effectiveness and a high safety profile^11^. Recent studies highlighted the importance of NMDA receptors in a variety of cellular activities, including those in the testes. These receptors participate in signaling pathways that control testicular development, spermatogenesis, and steroidogenesis. NMDA receptors are found in testicular cells, notably in Leydig and Sertoli cells, indicating that glutamate signaling might impact male reproductive health^12,13^. The overstimulation of NMDA receptors has been related to altered testosterone synthesis and sperm formation^14^. This finding is supported by data that NMDA receptor antagonists can improve various reproductive functions in animal models. The NMDA receptor is a ligand-gated cation channel in nerve cells that produces Ca2 + influx. Furthermore, NMDA receptors are expressed in the testes and affect sperm motility, germ cell differentiation, and apoptosis^15,16^. At the same time, the NMDA receptor interacts with the insulin signaling pathway, and abnormalities of the insulin signaling pathway are induced by RIS^17^.

Extracellular signal-regulated kinases (ERK) belong to the mitogen-activated protein kinase family, which plays a role in signaling cascades and transmits extracellular signals to intracellular targets^18^. ERK activation triggers oxidative stress through the generation of reactive oxygen species that can harm cellular components. This damage is especially alarming regarding testicular function, as it may result in diminished sperm quality, compromised steroidogenesis, and potential Leydig cell malfunction^19^. Additionally, ERK activation is closely related to insulin resistance associated with obesity and type 2 diabetes mellitus^20,21^.

Nuclear factor erythroid 2-related factor 2 (Nrf2), a ubiquitous transcription factor that affects oxidative stress response, is one of the key tissue-protective mechanisms. It regulates antioxidant, anti-inflammatory, and detoxifying genes. Furthermore, Nrf2 has been described as an important regulator of normal mitochondrial structure and function that is required for mitochondrial biogenesis, mitophagy, and mitochondrial integrity^22^.

The main objective of the current experiment was to evaluate the effect of MEM on testicular damage and insulin resistance induced by chronic RIS administration in rats. Furthermore, the possible role of the ERK1/2-Nrf2 pathway was investigated in the potential effects of MEM.

## Materials and methods

### Ethics

Our experimental protocol received permission from the Study Ethics Committee of the Faculty of Medicine, Minia University (Approval No.28:3/2021), and all methods were performed in accordance with the relevant scientific guidelines and regulations. The current study is reported in accordance with ARRIVE guidelines.

### Chemicals

RIS was purchased from Marcyrl Pharmaceutical Industries, Egypt. MEM was gained from Copad Egypt for trade and pharmaceutical industries, Egypt. Adiponectin was quantified by a Rat Adiponectin ELISA kit (MBS8244709) purchased from MyBioSource, San Diego, USA. Resistin was measured by the Rat ELISA Kit (ab289699), (Abcam, USA). Rat insulin ELISA Kit (E-EL-R3034) (Elabscience, USA) was used to measure fasting insulin levels. Serum levels of total and free testosterone were determined using the Rat Testosterone ELISA Kit (MBS282195), purchased from MyBioSource, USA. Anti-caspase-3, polyclonal antibody (ab184787) (abcam, USA). Activity assay kits for reduced glutathione (GSH), glutathione reductase (GR), glutathione peroxidase (GPx), glutathione S-transferase (GST) and catalase (CAT) were purchased from Elabscience, USA (Cat. No.: E-BC-K030-S, Cat. No.: E-BC-K099-S, Cat. No.: E-BC-K096-S, Cat. No.: E-BC-K278-S and Cat. No.: E-BC-K031-M, respectively). ELISA kits for interleukin-6 (IL-6), tumor necrosis factor-α (TNF-α) and nuclear factor-kappa B (NF-kB) were purchased from Elabscience, USA (Cat. No.: E-EL-R0015, Cat. No.: E-EL-R0019 and Cat. No.: E-EL-R0674, respectively).

### Animals and experimental design

Adult male Wister albino rats weighing 200–250 g were bought from the Animal House at Nahda University, Beni-Suef, Egypt. The rats were housed in plastic cages in a well-ventilated room of natural photoperiod of about 12:12 h light darkness cycle at temperature: 27 ± 10 OC and 40–50% relative humidity) as prescribed by the United States National Institute for Health^23^. Fed with rat chow and water ad libitum. The animals received humane care according to Care and Use of Laboratory Animals by the National Academy of Science and National Institute of Health and in compliance with ethical regulation of national and institutional guidelines for the protection of experimental animals’ right^24^.

Animals were randomized into six groups (6 rats/ group) as follows

Group I (Negative control): rats received 1 mL carboxymethylcellulose (CMC) for 4 weeks.

Group II (MEM-5): rats were given MEM (5 mg/kg/day, p.o.) for 4 weeks suspended in CMC^25^.

Group III (MEM-10): rats were administered MEM (10 mg/kg/day, p.o.) for 4 weeks suspended in CMC^26^.

Group IV (Positive control): rats were administered RIS (2.5 mg/kg/day, p.o.) for 4 weeks suspended in CMC^6^.

Group V (RIS + MEM-5): rats were given MEM (5 mg/kg/day, p.o.) suspended in CMC^25^, co-administered with RIS the same as in RIS group for 4 weeks^6^.

Group VI (RIS + MEM-10): rats were given MEM (10 mg/kg/day, p.o.) suspended in CMC^26^, co-administered with RIS the same as in RIS group for 4 weeks^6^.

### Sampling

Finally, rats were anesthetized with an IP injection of urethane (25% in a dosage of 1.6 gm/kg)^27^. Blood was drawn from the abdominal aorta and centrifuged for 15 min at 5000 rpm (Janetzki T30 centrifuge, Germany). The serum was then stored at -80 °C for biological examination. The testes were removed from each rat and rinsed with cold saline before being separated into sections for histopathological analysis; other portions were snap-frozen in liquid nitrogen, kept at -80 °C, and then homogenized in cold potassium phosphate buffer (pH 7.4) for different biochemical assays^28^.

### Biochemical assay

#### Assay of testicular oxidative stress and inflammatory markers

Testicular MDA, NOx, SOD, GSH, and CAT were measured using assay kits according to the manufacturer’s guidelines. Levels of IL-6, TNF-α, and NF-κB were detected using ELISA kits according to the manufacturer’s guidelines.

#### Assay of the serum biomarkers

Serum testosterone, resistin, and adiponectin concentrations were determined by commercial kits according to the manufacturer’s instructions.

#### Measurement of homeostatic model assessment (HOMA-IR) of insulin resistance

Fasting blood glucose and insulin levels were measured. The HOMA-IR equation was used to determine insulin resistance in rats. The Homeostasis Model Assessment (HOMA-IR) was determined by dividing the fasting glucose (mg/dl) and insulin (µIU/ml) concentrations by 405^29,30^.

### Western blotting analysis

Testis homogenates (50 g total proteins) were heated for 5 min in a loading buffer containing 2-mercaptoethanol before being loaded on a 12% sodium dodecyl sulfate-polyacrylamide gel electrophoresis (SDS-PAGE) running for 2 h at 100 V. Proteins were put on polyvinylidene fluoride (PVDF) membranes after electrophoresis. After blocking for 1 h in a Tris-buffered saline (TBS-T) blocking solution containing 5% (w/v) non-fat milk and 0.05% Tween-20, they were incubated with primary antibodies (1:1000) for rabbit anti-Nrf2 antibody, (1:1000, ab31163 abcam, Cambridge, UK) rabbit anti-ERK1 + ERK2 antibody (1:1000, ab54230, abcam), rabbit anti-ERK1 (phospho T202) + ERK2 (phospho T185) antibody (1:1,000, ab201015, abcam) and β-actin (Santa Cruz Biotechnology, Santa Cruz, CA) overnight at 4 °C. As a secondary antibody, a 1:5000 dilution of horseradish peroxidase-conjugated polyclonal goat anti-rabbit immunoglobulin (Cell Signaling Technology Inc., MA, USA) was employed in the blocking buffer. The immunoreactive proteins were quantified using the Chemiluminescence kit (GE Healthcare, Little Chalfont, UK) and a luminescent image analyzer (LAS-4000, Fujifilm Co., Tokyo, Japan), following the manufacturer’s instructions. Densitometric analysis was conducted using the Image Processing and Analysis Java (ImageJ, 1.8.0_172) program. Data were acquired in proportion to the control group after normalization to the equivalent amounts of β-actin^31^.

### Histopathology study

The testes were placed in Bouin’s solution before being embedded in paraffin. A microtome was used to cut 5 μm thick cross-sections. The slices were stained with hematoxylin and eosin, and examined under an optical microscope. Testicular injury was assessed by a semiquantitative analysis for tubular degeneration, necrosis, and germinal epithelium integrity using a scale from 0 to 4, where 0 means no abnormal findings, and 4 means severe abnormal findings^32^.

### Immunohistochemical examination

The tissue blocks were sectioned into 5 μm sections and mounted on positively charged slides for immunohistochemistry with caspase 3. Sections were dewaxed, and endogenous enzymes were inactivated with 3% H_2_O_2_. The primary antibody was applied and incubated overnight at 4 °c. Rinsing with PBS solution was done. A secondary antibody was added to sections and incubated for 30 min. After that, samples were rinsed with PBS solution, and the DAP color development system was performed for 5 min. After dehydration, the slides were sealed with DPX and examined microscopically. Staining results were evaluated according to the degree and percentage of positively stained cells. Sections exhibiting no color, faint yellow, yellow brown and dark brown were recorded as 0, 1, 2, and 3, respectively. Under the high power of magnification of the microscope (x400), the percentage of positive cells was determined to be < 5% of cells, 5–25% of cells, 26–50% of cells, and > 50% of cells, which were recorded as 0, 1, 2, and 3 respectively. The two scores were added, and the expression was considered positive if the total score was ≥ 3 points^33,34^.

### Statistical analysis

All parameters were presented as means ± standard error of the mean (SEM). The data were analyzed using one-way analysis of variance (ANOVA), followed by the Tukey-Kramar post-analytic test. P values less than 0.05 were deemed significant. For statistical computations, GraphPad Prism was used (version 5.01 for Windows, GraphPad Software, San Diego, California, USA, and www.graphpad.com).

## Results

### Effect of MEM on fasting glucose, fasting insulin, and HOMA-IR

**Fig. 1:**
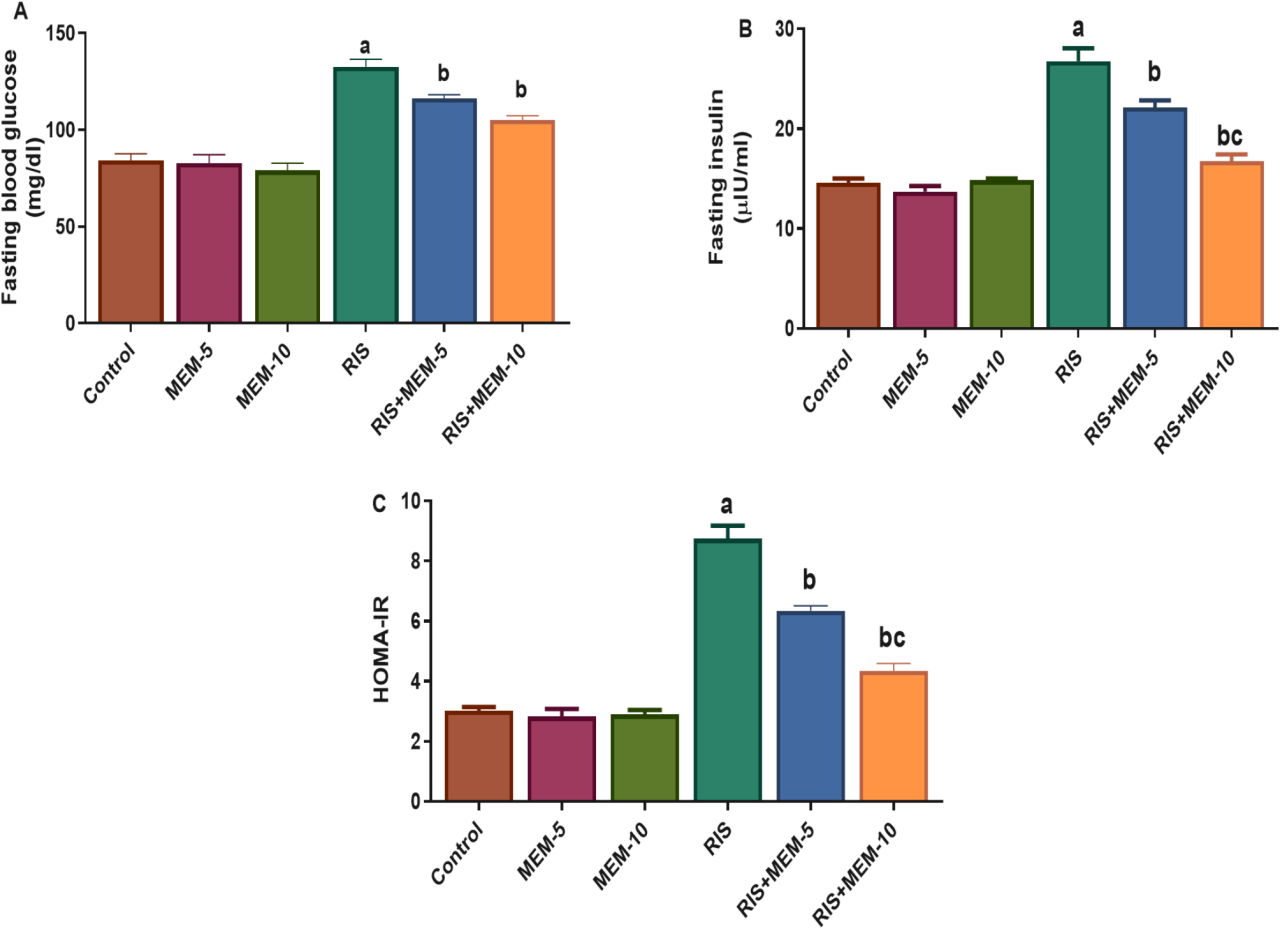
Effect of MEM on fasting glucose (**A**), fasting insulin (**B**) and HOMA-IR (**C**). Results represent the mean ± SEM (*n* = 6). Significance is at *p* < 0.05. ^a^significant difference relative to control group; ^b^significance difference with respect to RIS group; ^c^significance difference against RIS + MEM-5 group.

In the RIS group, fasting glucose, fasting insulin, and HOMA-IR were increased relative to the control group. In contrast, MEM, when co-administered with RIS, reduced these three parameters relative to the RIS group in a dose-dependent manner (Fig. 1). These findings indicate that MEM has a protective impact against RIS-evoked insulin resistance.

### Effect of MEM on resistin and adiponectin serum levels

**Fig. 2:**
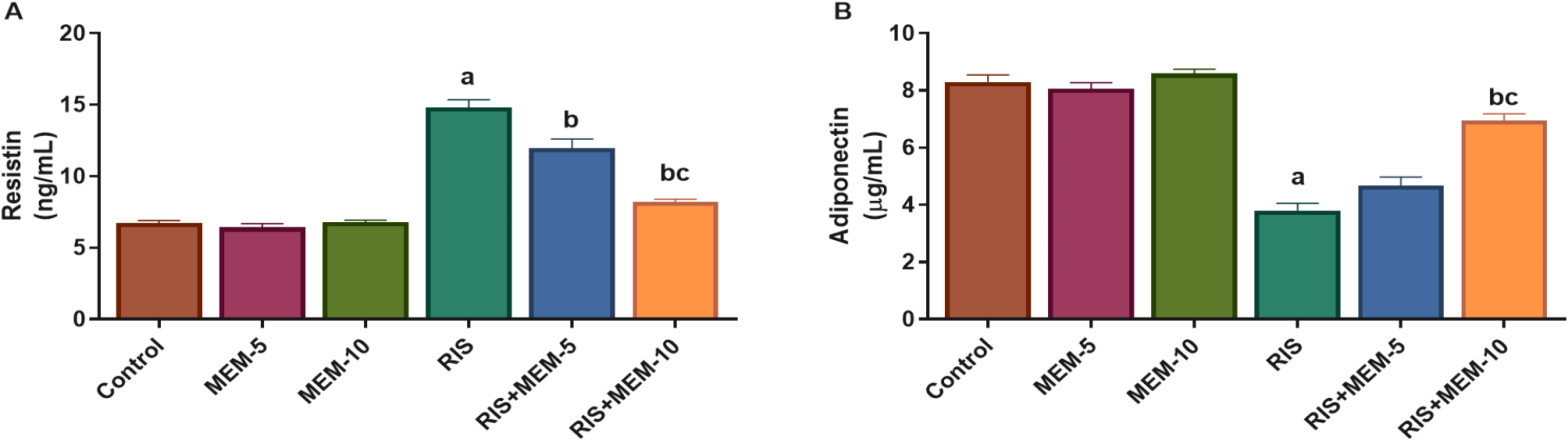
Effect of MEM on serum resistin (**A**) and adiponectin (**B**). Results represent the mean ± SEM (*n* = 6). Significance is at *p* < 0.05. ^a^significant difference relative to control group; ^b^significance difference with respect to RIS group; ^c^significance difference against RIS + MEM-5 group.

In the RIS group, serum resistin levels increased while adiponectin levels decreased when compared to the control group. In the RIS + MEM-5, and RIS + MEM-10 groups, resistin was reduced, and adiponectin was increased relative to the RIS group in a dose-dependent manner (Fig. 2). These results show that MEM has a powerful impact on restoring hormonal abnormalities induced by RIS.

### Effect of MEM on serum testosterone levels

**Fig. 3:**
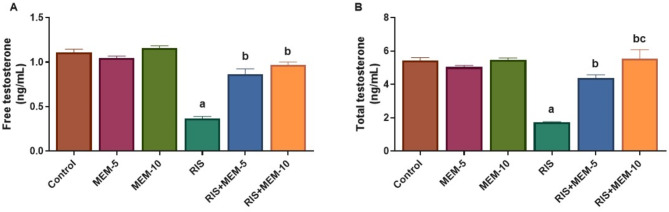
Effect of MEM on both free testosterone (**A**) and total testosterone (**B**) serum levels. Results represent the mean ± SEM (*n* = 6). Significance is at *p* < 0.05. ^a^significant difference relative to control group; ^b^significance difference with respect to RIS group; ^c^significance difference against RIS + MEM-5 group.

Free and total testosterone was reduced with RIS, relative to the control group. In RIS + MEM-5, and RIS + MEM-10 groups, free and total testosterone levels were increased relative to the RIS group **[**Figure 3**].** The results presented here indicate that MEM significantly influences the restoration of reduced testosterone levels caused by RIS.

### Effect of MEM on testicular MDA, NOx, SOD, GSH and CAT levels

MDA and NOx were elevated in the RIS group, while SOD, CAT activities, and GSH levels were significantly decreased, as compared to the control group. In contrast, MDA and NOx parameters were significantly reduced in MEM-treated groups whereas activities of SOD, CAT, and GSH levels were significantly increased when compared to the RIS group (Table 1). The findings demonstrate that MEM considerably reduced RIS-induced oxidative stress and restored antioxidant capability.

**Table 1:** Effect of MEM on testicular MDA, NOx, SOD, GSH and CAT levels.

Group	MDA (mmol/g tissue)	NOx (nmol/g tissue)	SOD (U/g tissue)	GSH (mg/g tissue)	CAT (U/g tissue)
Control	13.4 ± 0.67	15.50 ± 0.90	40.40 ± 0.66	112.8 ± 2.15	139.5 ± 4.28
MEM-5	13.0 ± 1.02	14.4 ± 1.05	40.53 ± 0.38	111.5 ± 2.40	137.30 ± 4.32
MEM-10	11.77 ± 0.54	16.94 ± 1.38	40.17 ± 0.57	112.8 ± 2.94	140.20 ± 5.93
RIS	38.73 ± 2.99^a^	40.2 ± 2.35^a^	19.94 ± 0.70^a^	68.83 ± 2.61 ^a^	87.67 ± 2.84 ^a^
RIS + MEM-5	30.36 ± 2.31^b^	32.85 ± 0.84^b^	23.60 ± 1.32^b^	79.50 ± 3.08	96.67 ± 3.35
RIS + MEM-10	20.84 ± 1.47^bc^	22.91 ± 1.48^bc^	31.13 ± 0.47^bc^	92.83 ± 3.12^bc^	117.50 ± 4.07 ^bc^

Results represent the mean ± SEM (*n* = 6). Significance is at *p* < 0.05. ^a^significant difference relative to control group; ^b^significance difference with respect to RIS group; ^c^significance difference against RIS + MEM-5 group.

### Effect of MEM on testicular inflammatory mediators

The RIS group showed a significant rise in testicular IL-6, TNF-α, and NF-ĸB, as compared to the control group. In contrast to the findings of the RIS group, there was a notable decrease observed in IL-6, TNF-α, and NF-ĸB in the MEM-treated groups, as compared to RIS group (Table 2). The findings indicate that MEM’s capability to alleviate the upregulated testicular inflammatory mediators evoked by RIS.

**Table 2:** Effect of MEM on testicular inflammatory mediators.

Group	IL-6 (Pg/g tissue)	TNF-α (Pg/g tissue)	NF-KB (Pg/g tissue)
Control	14.60 ± 0.79	60.04 ± 2.07	99.58 ± 3.08
MEM-5	13.67 ± 0.72	58.99 ± 2.54	97.42 ± 6.63
MEM-10	13.44 ± 0.63	62.97 ± 2.17	100.20 ± 3.28
RIS	34.33 ± 1.58^a^	112.70 ± 3.73^a^	304.50 ± 7.00 ^a^
RIS + MEM-5	23.35 ± 1.26^b^	99.83 ± 1.59^b^	227.70 ± 7.15 ^b^
RIS + MEM-10	18.33 ± 0.70^bc^	75.73 ± 2.41^bc^	147.30 ± 3.98 ^bc^

Results represent the mean ± SEM (*n* = 6). Significance is at *p* < 0.05. ^a^significant difference relative to control group; ^b^significance difference with respect to RIS group; ^c^significance difference against RIS + MEM-5 group.

### Effect of MEM on testicular ERK1/2 and Nrf2 expression

**Fig. 4:**
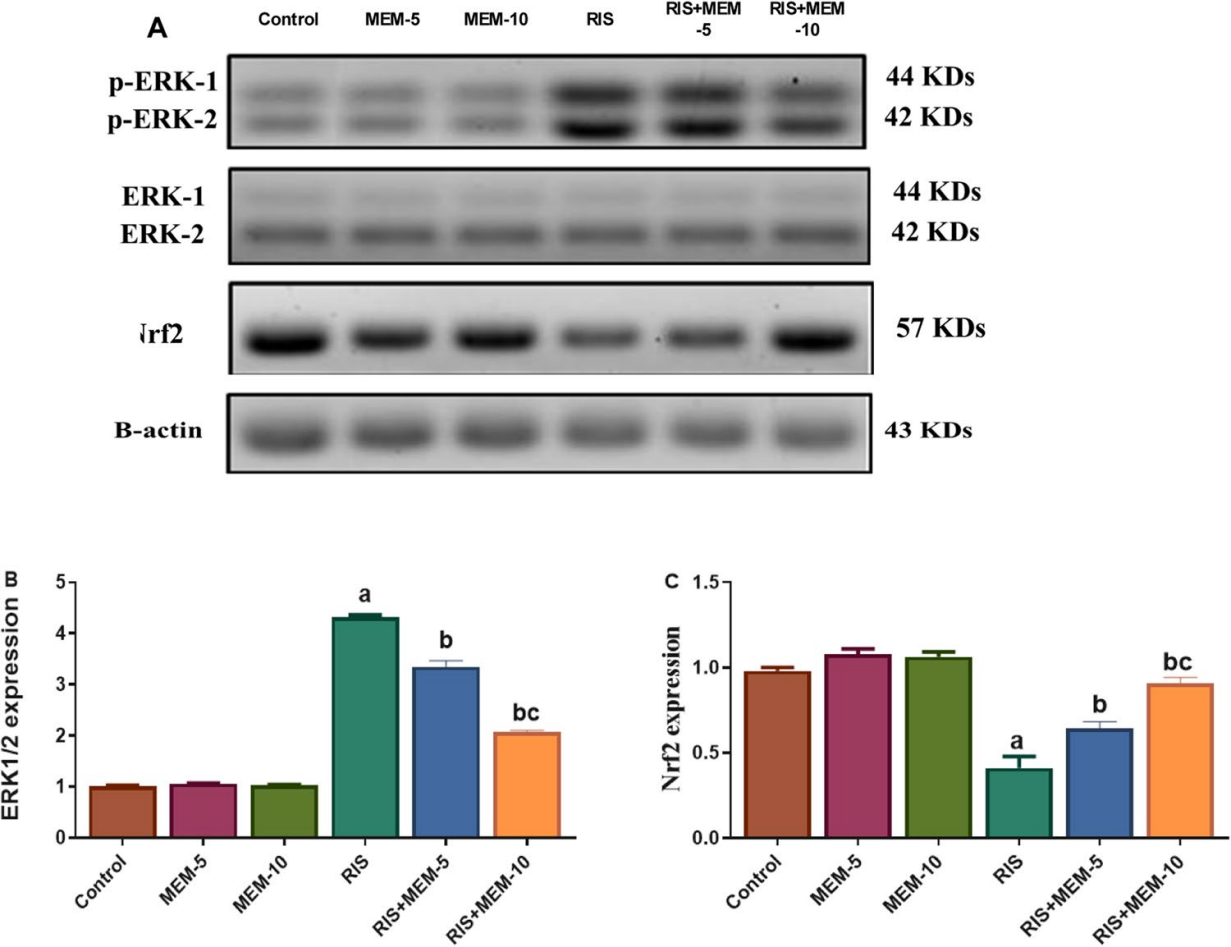
Representative western blot bands (**A**), and protein expression levels of testicular ERK1/2 (**B**), and Nrf2 (**C**). Results represent the mean ± SEM (*n* = 3). Significance is at *p* < 0.05. ^a^significant difference relative to control group; ^b^significance difference with respect to RIS group; ^c^significance difference against RIS + MEM-5 group.

In the RIS group, expression of ERK1/2 was increased, whereas Nrf2 expression was reduced as compared to the control group. In the MEM-treated groups, the reverse occurred, as expression of ERK1/2 was reduced and Nrf2 expression was increased, as compared to the RIS group (Fig. 4). The results indicate that MEM upregulates the testicular Nrf2 expression and downregulates testicular ERK1/2 expression.

### Effect of MEM on histopathological aberrations of rat testes

**Fig. 5:**
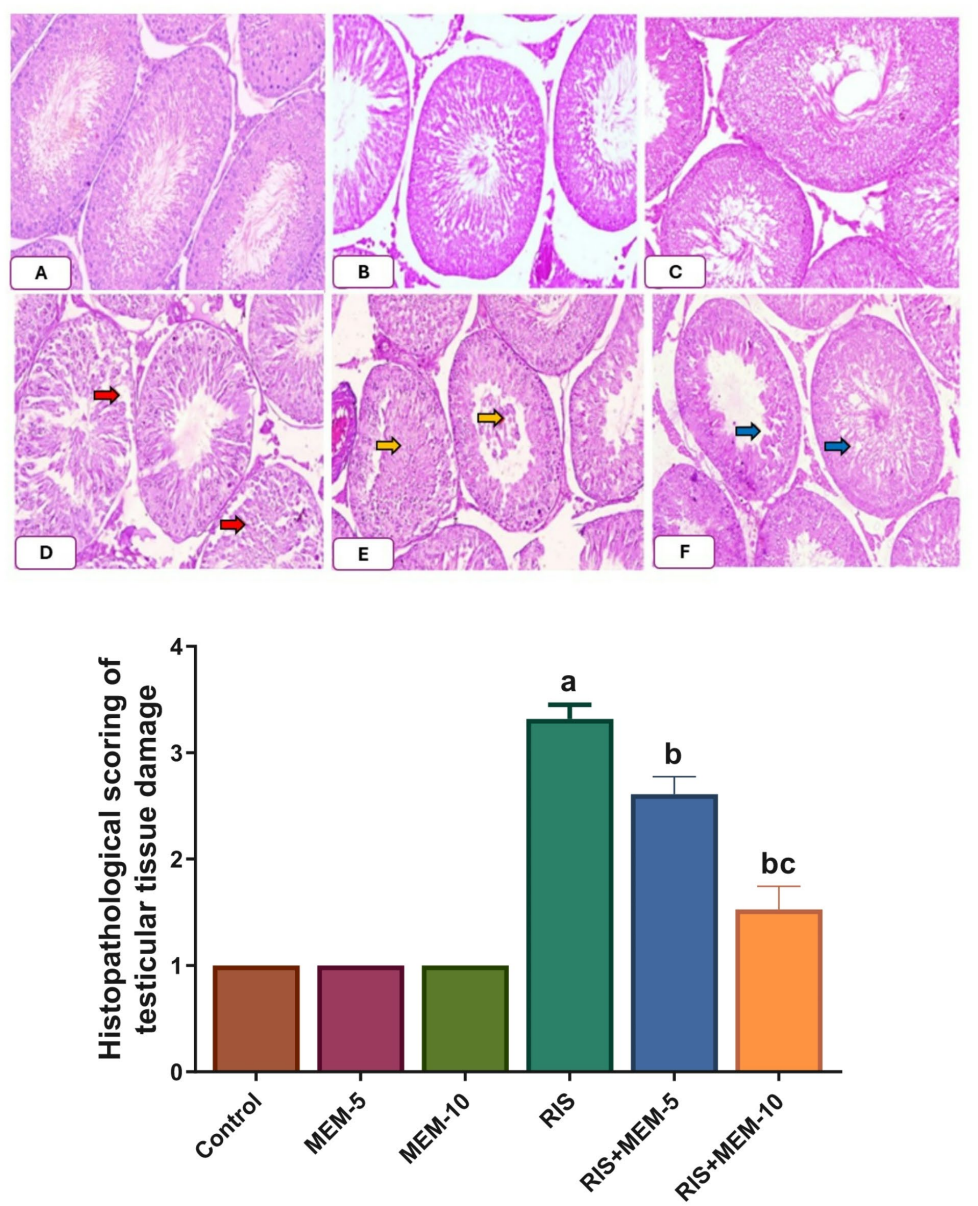
Effect on histopathological picture of testicular tissue (x200) and scoring of testicular damage. (**A**, **B**, **C**); control, MEM-5, and MEM-10 groups respectively, showed normal seminiferous tubules, and normal germinal epithelium with normal interstitial cells of Leydig. (**D**): RIS group, red arrows refer to necrosis of both superficial and deep layers of the germinal epithelium lining closely packed seminiferous tubules. These tubular lumens were filled with amorphous material containing cellular debris. (**E**): RIS + MEM-5 group; yellow arrows refer to necrosis of superficial and deep layers of the germinal epithelium lining closely packed seminiferous tubules with the destruction of its basement membrane. These tubular lumens were filled with amorphous material containing cellular debris. (**F**): RIS + MEM-10 group; blue arrows refer to injury limited to superficial germinal layer in few seminiferous tubules, however, most seminiferous tubules are normal with normal germinal epithelium and Interstitial cells of Leydig are also normal. Results represent the mean ± SEM (*n* = 6). Significance is at *p* < 0.05. ^a^significant difference relative to the control group; ^b^significance difference with respect to RIS group; ^c^significance difference against RIS + MEM-5 group.

In the control, MEM-5, and MEM-10 groups, normal testicular tissue with an orderly arrangement of germinal epithelium and intact seminiferous tubules was revealed. On the other hand, RIS group examinations revealed necrosis of all layers of germinal epithelium lining closely packed seminiferous tubules. These tubular lumens were filled with cellular debris-containing amorphous material. In addition, certain places showed the loss of tubular structures. Examination of the RIS + MEM-5 and RIS + MEM-10 groups exhibited disorganized, non-cohesive germinal cells as well as densely packed seminiferous tubules. Some tubules revealed necrosis limited only to the superficial layer of germinal cells, while other ones were lined by normal germinal epithelium in the RIS + MEM-10 group **[**Figure 5**].** MEM restored histopathological aberrations induced by RIS.

### Effect of MEM on immune expression of caspase-3 in rat testes

**Fig. 6:**
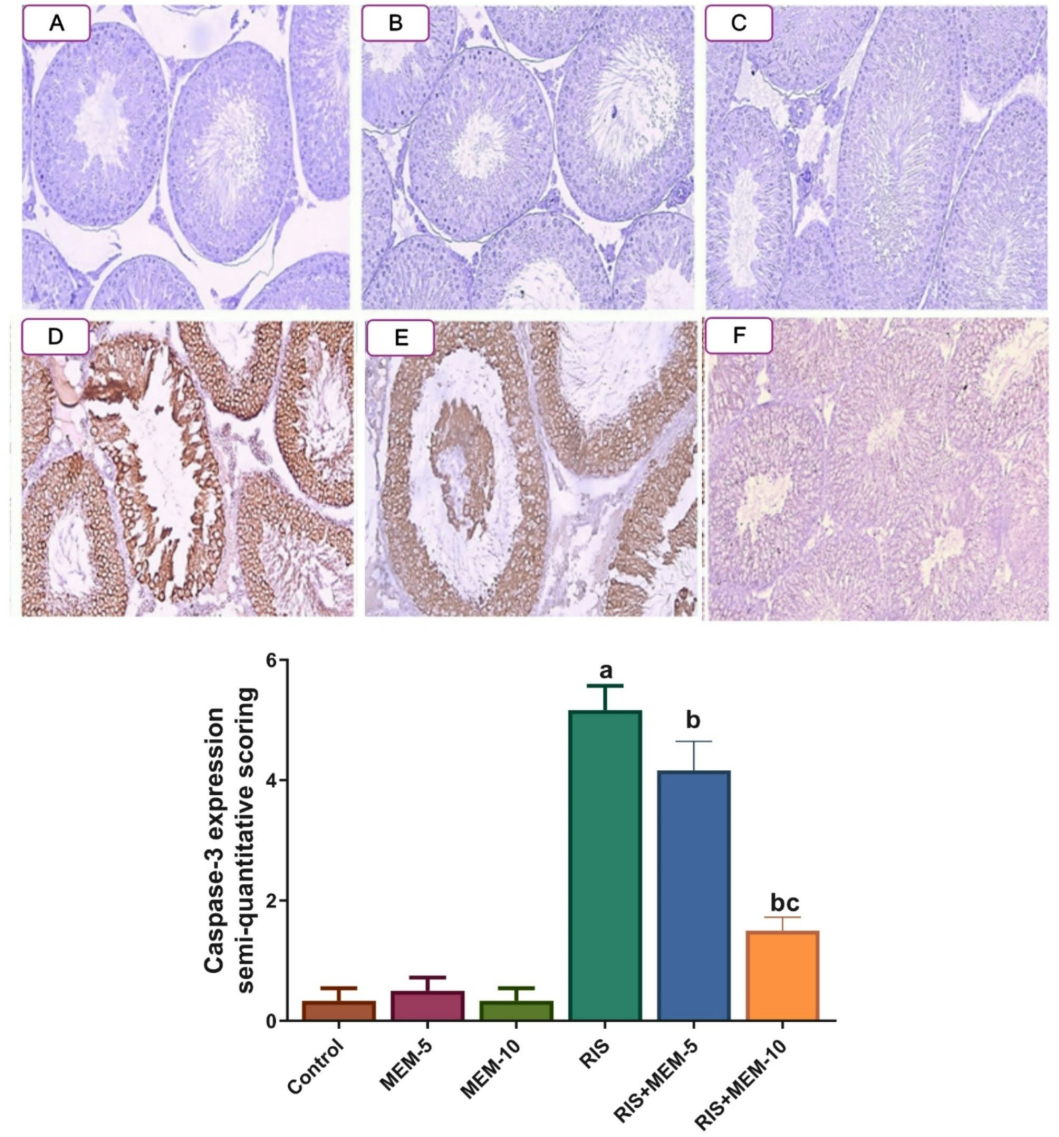
Effect of MEM on caspase-3 immunoexpression in testicular tissue (x200), and its semi-quantitative scoring. (**A**, **B**, **C**); control, MEM-5 and, MEM-10 groups respectively, show negative expression of caspase-3. (**D**,** E**): RIS and RIS + MEM-5 groups, respectively, show strong expression of caspase 3. (**F**): RIS + MEM-10 group; show weak expression of caspase-3. Results represent the mean ± SEM (*n* = 6). Significance is at *p* < 0.05. asignificant difference relative to control group; bsignificance difference with respect to RIS group; csignificance difference against RIS + MEM-5 group.

In the control, MEM-5, and MEM-10 groups, a negative expression of caspase-3 was shown. In the RIS group, a strong expression of caspase-3 occurred, which was evidenced by an increase in semi-quantitative scoring, relative to the control group. In contrast, in the RIS + MEM-5, and RIS + MEM-10 groups, a weak expression of caspase-3 was noticed in a dose-dependent manner, confirmed by a significant reduction in semi-quantitative scoring relative to the RIS group (Fig. 6). Our findings highlight that MEM-alleviated testicular apoptosis induced by RIS via the downregulation of testicular caspase-3 expression.

## Discussion

In addition to cognitive impairment, 90% of dementia patients have behavioral and psychological manifestations such as psychosis, violence, agitation, and depression. Inpatient hospitalization, cognitive impairment, and costs of care are all exacerbated by dementia-related psychosis (DRP), which includes delusions and hallucinations. Delusions and hallucinations tend to worsen with the length and severity of the illness, though there are individual differences^35^. DRP mechanisms are multifaceted, including several neurological components. Furthermore, diabetes mellitus is a chronic condition that affects around 6% of the general population and can lead to consequences such as neuropathy. Diabetes is frequently associated with schizophrenia. There are two basic explanations for this phenomenon. People with diabetes have a greater frequency of mental illnesses, and antipsychotics can induce metabolic irregularities. In schizophrenia, the risk is moderate for risperidone^36^. Recent studies demonstrated a notable correlation between schizophrenia and an elevated chance of subsequently acquiring dementia. Those with schizophrenia are over twice as likely to acquire dementia compared to those without the disease^37,38^. This point justifies the benefit of using risperidone in combination with memantine.

The present study aimed to evaluate the prophylactic potential role of MEM in attenuating insulin resistance in prolonged risperidone treatment with concurrent testicular dysfunction associated with insulin resistance and metabolic disturbance.

Aiming to find a link between testicular damage and long-term use of RIS and the subsequent effect of MEM; markers for testicular function (serum testosterone), IR (HOMA-IR, adiponectin, and resistin), inflammatory status (IL-6, TNF-α, and NF-kB) and oxidative stress, and also expression of ERK1/2 and Nrf2 were determined.

Reactive oxygen species (ROS) are known to have an important impact on testicular tissue damage. Lipid peroxidation has also been linked to a decrease in membrane integrity and function as well as the induction of apoptosis in seminiferous tubules. Lower glutathione levels are strongly correlated with important biological processes, including drug detoxification, the destruction of intracellular peroxides and free radicals, and the regulation of cellular oxidation-reduction. Glutathione level reductions in cells are therefore regarded as a sign of oxidative stress. It has been demonstrated that RIS increases lipid peroxidation, ROS production, mitochondrial membrane potential collapse, GSH depletion, and lysosomal membrane damage in nonreproductive cells (liver and kidney)^39–41^. So, we investigated major markers of oxidative stress, such as MDA, SOD, CAT, and GSH to find out the role of oxidative stress in possible reproductive toxicity induced by RIS.

In the current study, RIS induced testicular damage alongside insulin resistance. Testicular damage was supported by the reduction in serum testosterone. At the same time, RIS induced oxidative stress as shown by a reduction of GSH, CAT, and SOD and an increase of MDA and NOx. RIS also induced apoptosis as evidenced by an increase in the apoptotic marker caspase 3. RIS-induced inflammatory status in testis as evidenced by an increase in IL-6, TNF-α, and NF-kB. This is in agreement with previous reports that stated the induction of inflammation, oxidative stress, and apoptosis with RIS in many tissues^42–44^. These changes run parallel to the severe testicular damage shown in the histopathological examination of rat testes and the histopathological scoring of the degree of tissue damage.

On the other hand, IR occurred with RIS and was confirmed with elevated fasting glucose, fasting insulin, and HOMA-IR. For further confirmation of IR, resistin; an indicator of insulin resistance; and adiponectin; an indicator of insulin sensitivity^45^; were measured, and we found that resistin was increased and adiponectin was reduced, which are highly sensitive indicators for IR occurrence and reduction of insulin sensitivity^45^.

A large number of studies have documented correlations between the use of anti-dopaminergic antipsychotic medications and untoward consequences, specifically abrupt weight gain, metabolic disruptions, and endocrine irregularities. Subsequently, obesity and hyperinsulinemia result in a decrease in the production of testosterone in the testis. Additionally, it has been documented that risperidone induces insulin resistance, which is accompanied by an elevation in resistin levels. Furthermore, a reduction in adiponectin levels has been positively correlated with weight gain and fat mass^46^.

Co-administration of MEM with RIS attenuated testicular damage, which was evidenced by the increase in serum testosterone, antagonizing oxidative stress status in the testis, and the improvement of the histopathological picture of the testis. This improvement of testicular injury is accompanied by antagonizing IR that is shown through a reduction of fasting insulin, fasting glucose, HOMA-IR, and resistin and an increase in adiponectin. Many functional and structural abnormalities and consequences in the testis are caused by IR, involving male reproductive disorders, impaired testicular function, spermatogenesis, sperm count, sperm motility, diminished seminal fluid volume, and testosterone levels^47^. Therefore, MEM mostly antagonized gonadotoxicity through the amelioration of IR induced by RIS. This finding runs parallel to previous reports about the inverse relation between MEM administration and the occurrence of IR^48,49^.

Running in the same direction, it was reported that NMDA receptor is highly distributed in testis^50^ which run parallel to other reports stated that NMDA activation may induce oxidative stress in many organs^51,52^.

In addition, MEM was reported in previous studies that it has a direct anti-oxidant action by blocking NMDA receptors in many organs including CNS^53^, liver^54^, stomach^55^ and kidney^56^. Surprisingly, Huang and co-workers reported that the development of tissue damage occurs in diabetes is related to an increase in glutamate release that aggravates β-cells dysfunction by excessive stimulation of NMDA receptors on β-cells, leading to activation of oxidative stress^57^. In light of the previous findings, it can be suggested that MEM by blocking NMDA receptors, antagonized the oxidative stress and its deteriorating effect occurred with RIS-induced prolonged hyperglycemia.

Increasing evidence indicates that the activation of ERK appears to be a contributing factor in the development of insulin resistance induced by oxidative stress in other tissues^58^. In various conditions, the induction of insulin resistance can be observed through the activation of ERK1/2, triggered by elevated levels of glucose^59,60^. A recent study that showed that deficiency in the ERK1/2 protects leptin-deficient mice from insulin resistance, further confirms the causative of ERK1/2 activation in the insulin resistance^61^.

In addition, numerous comprehensive investigations have been conducted to establish the strong correlation between oxidative stress and its involvement in aggravated insulin resistance, resulting in metabolic disturbances and their subsequent consequences in multiple organ dysfunctions, especially with second-generation antipsychotics like RIS^62,63^. From these previous interesting studies, it was revealed that the downregulation of Nrf2 activity via ERK1/2 stimulation contributes to oxidative stress-induced insulin resistance. Based on information from recent literature, this study was designated to explore a causal link between oxidative stress and insulin resistance with a focus on the regulatory role of the ERK1/2-Nrf2 pathway. In light of the previous data, ERK1/2 is implicated in the development of insulin resistance associated with obesity with dysregulation of adipocytokine expression and increased lipolysis activity. We have now examined the potential of pharmacological targeting of the ERK pathway for the treatment of testicular damage associated with insulin resistance. In the current work, ERK1/2 is upregulated with RIS and downregulated in the MEM + RIS group; however, the reverse occurs with Nrf2, which decreased with RIS and increased when MEM was co-administered with RIS.

It was reported that Nrf2 controls the expression of key components of the antioxidant system, such as glutathione and thioredoxin, and regulates the transcription of many ROS-detoxifying enzymes such as glutathione peroxidase, heme oxygenase, and several glutathione S transferases^64,65^.

As a result, in the current work, Nrf2 might play a critical role in IR development under oxidative conditions because insulin stimulates Nrf2, which could be boosted by inhibiting ERK1/2, showing that insulin stimulates Nrf2, which is negatively controlled by ERK. Similar studies found that forcing Nrf2 activation dramatically reduced insulin-induced ERK activity, which is thought to minimize oxidative stress and apoptosis-induced ERK activity while increasing insulin-mediated glucose uptake and antagonizing IR. As a result, our findings not only show that Nrf2 plays an important role in maintaining insulin sensitivity, but also suggest that Nrf2 may be important in the control of IR-mediated tissue damage. These findings add to previous evidence linking Nrf2 depletion to oxidative stress, apoptosis, and insulin resistance in various cells and organs^66,67^.

In agreement with current results, it was reported that NMDA receptor activation mediated sustained activation and extranuclear retention of the active ERK^68^. Furthermore, it was reported that NMDA inhibition increased Nrf2 expression^69^ and in other reports, NMDA modulation was associated with Nrf2 upregulation^70^. These findings highlight the effect of MEM as an NMDA antagonist when combined with RIS on ERK1/2 and Nrf2 expression with consequent anti-oxidant, anti-apoptotic effects that confer a protective on testicular tissue.

Surprisingly, MEM co-administration with RIS confers protection against testicular injury and attenuates all biochemical and histopathological abnormalities induced by RIS in a dose-dependent manner.

Nonetheless, the study has certain limitations that warrant future examination, including the limited sample size, the need for additional research to confirm the long-term effects of memantine, and the exploration of possible alternative mechanisms that may contribute to the protective effects of MEM.

## Conclusion

**Fig. 7:**
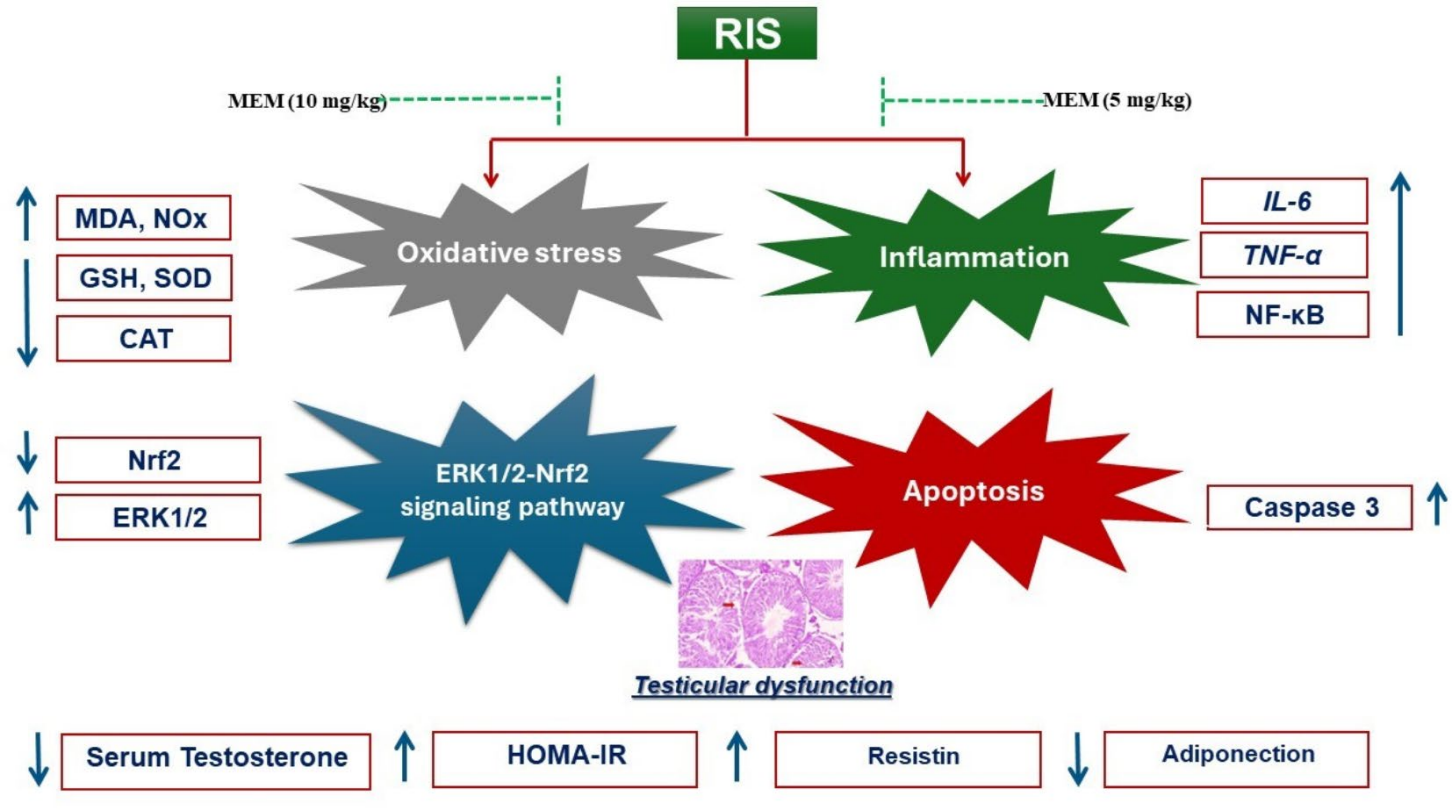
Graph outlining the mechanism of RIS-evoked testicular dysfunction and the potential protective impact of MEM. One of the authors, Ehab E. Sharata, used Microsoft PowerPoint to create this graph.

The current study provided several findings regarding the impact of insulin sensitivity and testicular damage induced by chronic RIS administration in rats. First, IR through oxidative damage, inflammation, and apoptosis may be a causal factor in the development of testicular injury in response to RIS treatment. Second, ERK is a negative regulator that causes RIS-induced oxidative cell damage, inflammation, and apoptosis. Third, ERK-mediated Nrf2 activity reduction is connected to oxidative damage, inflammation, and apoptosis-induced IR. Fourth, activating Nrf2 inhibits ERK function, restores oxidative stress-induced insulin resistance, and antagonizes inflammation. Finally, MEM can prevent oxidative stress, inflammation, and apoptosis in the testis that was induced by chronic RIS administration through ERK stimulation and Nrf2 suppression in a dose-dependent manner. These findings show that the ERK1/2-Nrf2 pathway may play an important role in MEM’s protective impact against testicular tissue damage caused by RIS and that targeting this pathway may provide a new therapeutic strategy for the treatment of testicular damage caused by insulin resistance. As demonstrated in (Fig. 7). Moreover, these unique findings require validation through clinical trials employing diverse dosages and durations of MEM administration to thoroughly assess the therapeutic efficacy of MEM in alleviating RIS-induced testicular damage.

## Electronic supplementary material

Below is the link to the electronic supplementary material.

Supplementary Material 1.

## Data Availability

The datasets used and/or analysed during the current study are available from the corresponding author on reasonable request.

## Abbreviations

MEM: Memantine
RIS: Risperidone
NMDA: *N*-Methyl-D-aspartate
CMC: Carboxymethylcellulose
MDA: Malondialdehyde
NOx: Total nitrites
SOD: Superoxide dismutase
HOMA-IR: Homeostatic model assessment of insulin resistance
Nrf2: Nuclear factor erythroid 2–related factor 2
ERK: Extracellular signal-regulated kinases
DRP: Dementia-related psychosis
