# Supplementary material for: Memantine abrogates testicular dysfunction induced by risperidone in rats with a potential role of ERK1/2-Nrf2-caspase-3 signaling pathway
Source: Sci Rep. 2025 Apr 15;15:12914. doi: 10.1038/s41598-025-94760-1 (PMC12000432; doi:10.1038/s41598-025-94760-1)
Supplement: Supplementary file 1 — Supplementary Material 1. [file 41598_2025_94760_MOESM1_ESM.pdf]

p-ERK 1,2

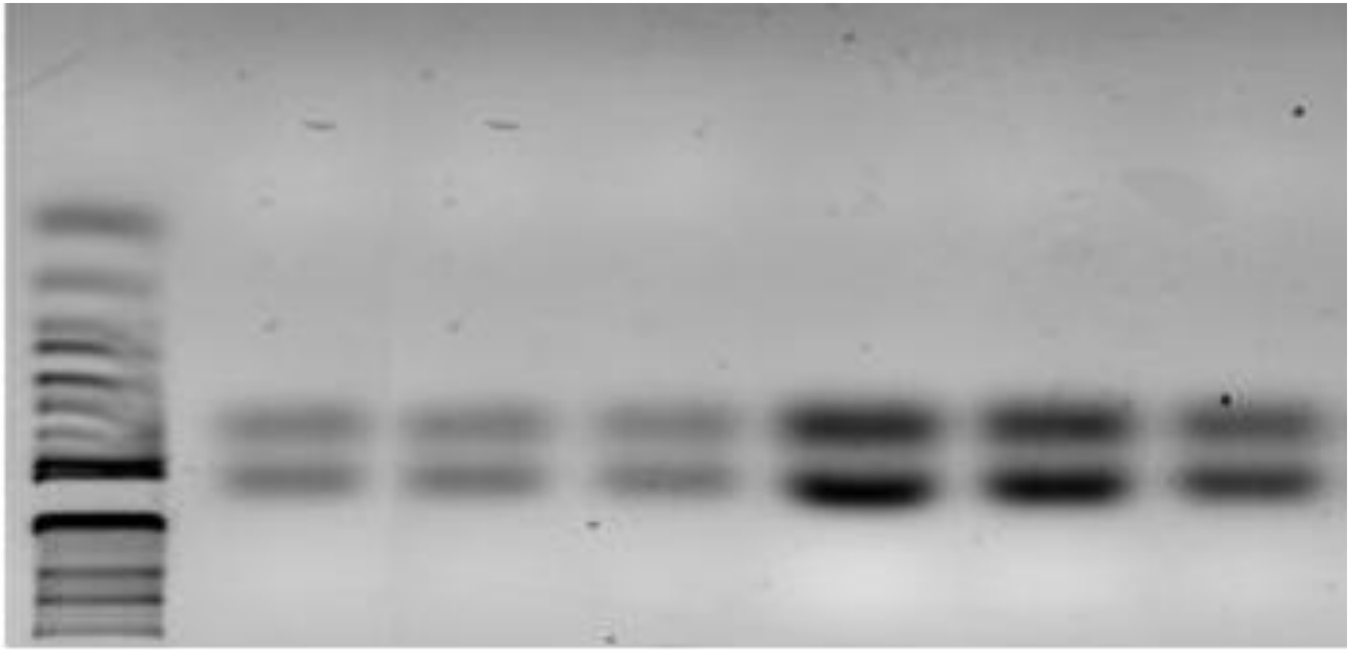

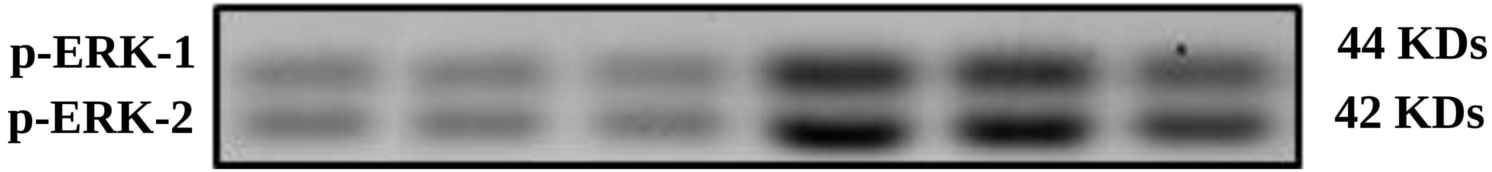

p-ERK 1,2

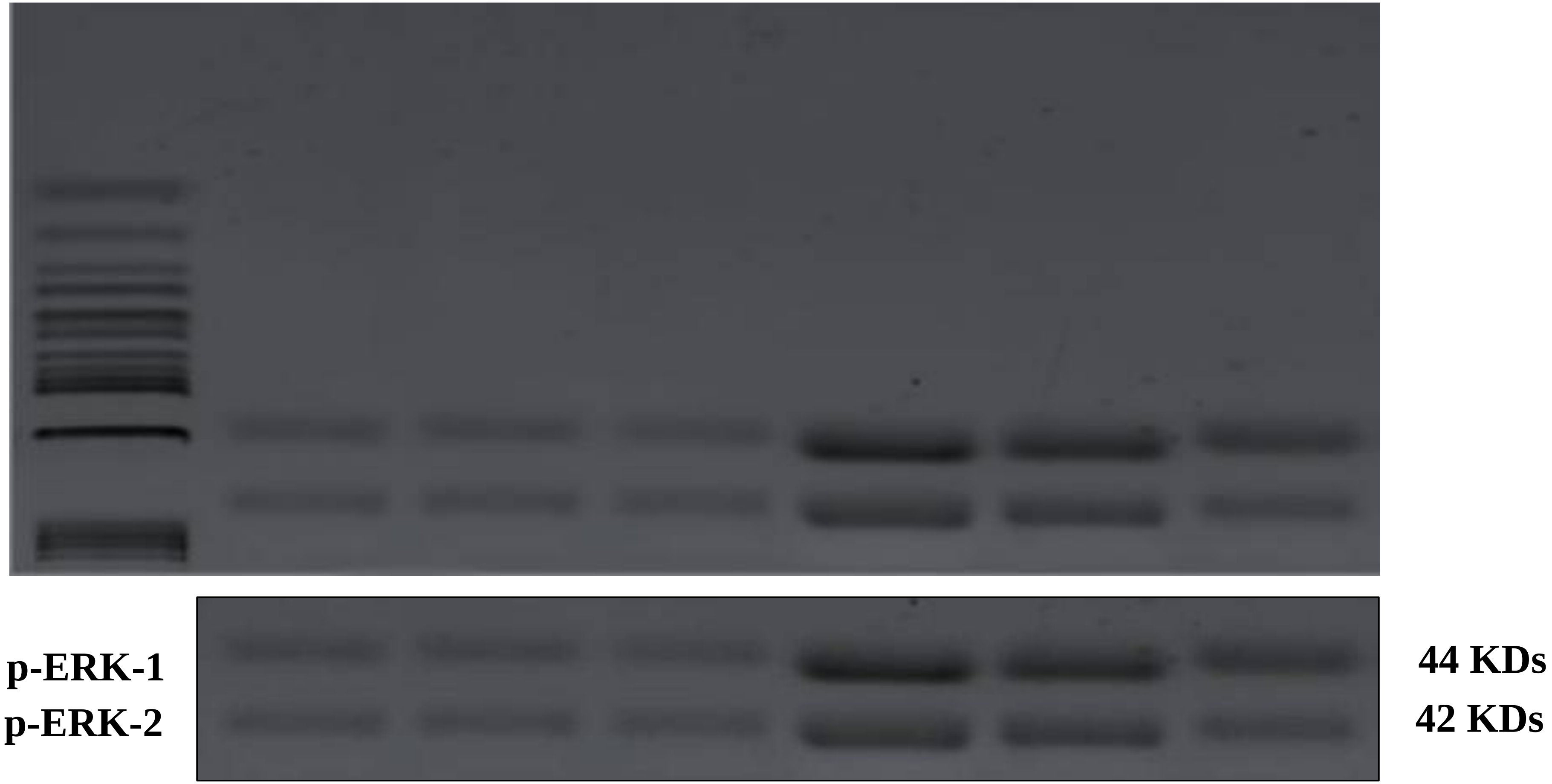

p-ERK 1,2

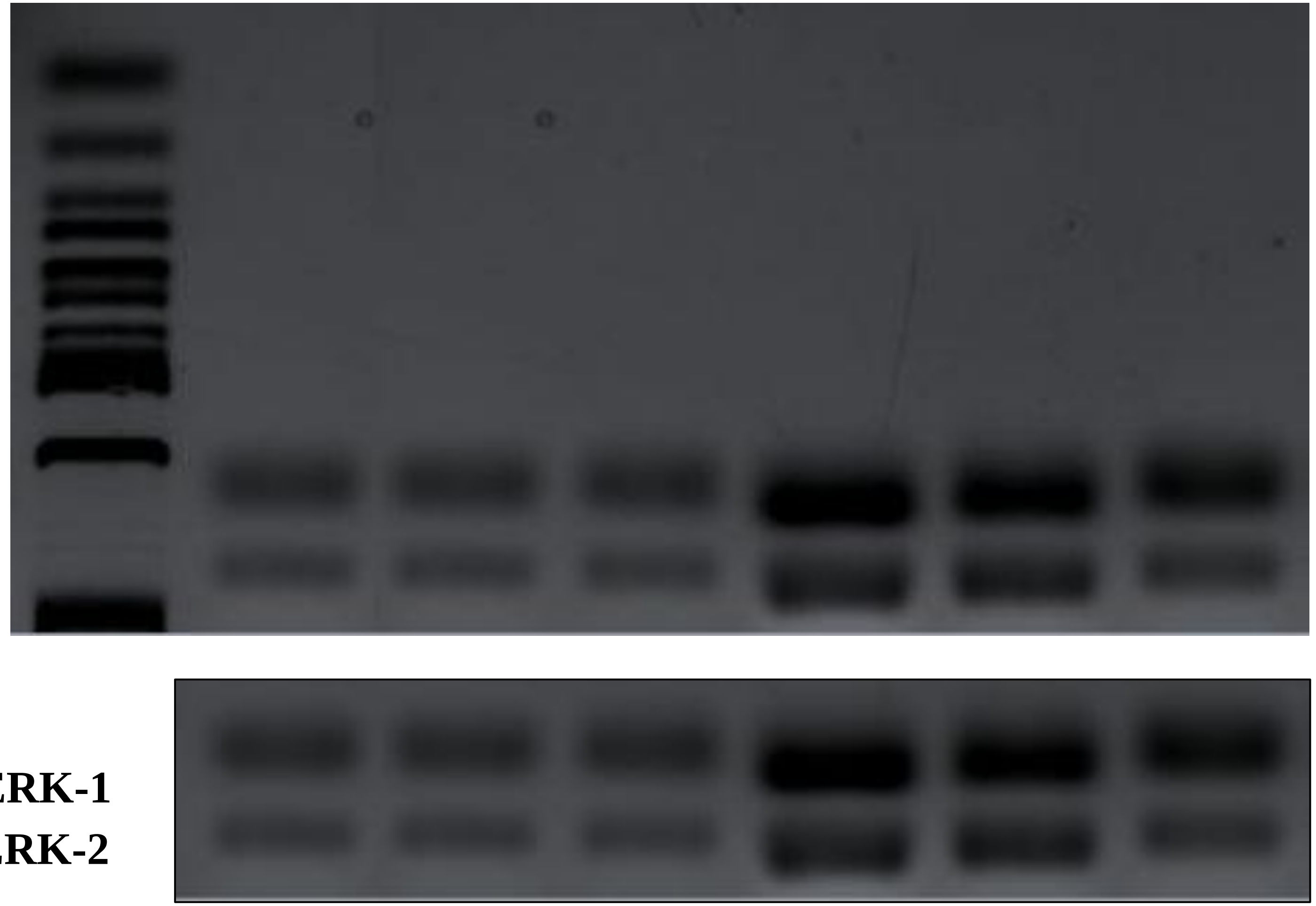

ERK 1,2

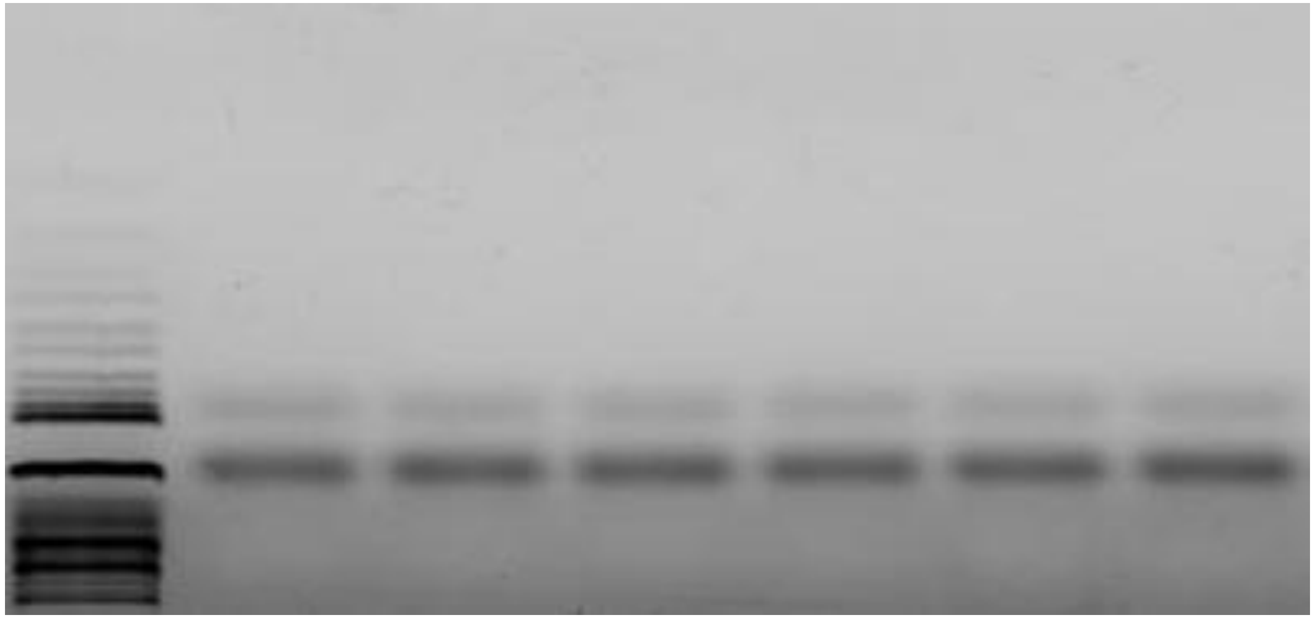

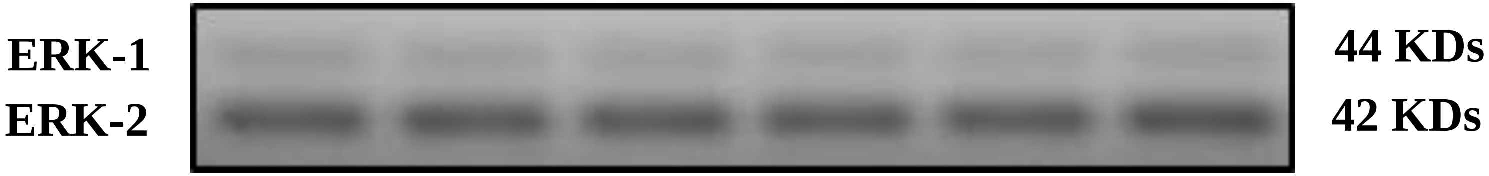

ERK 1,2

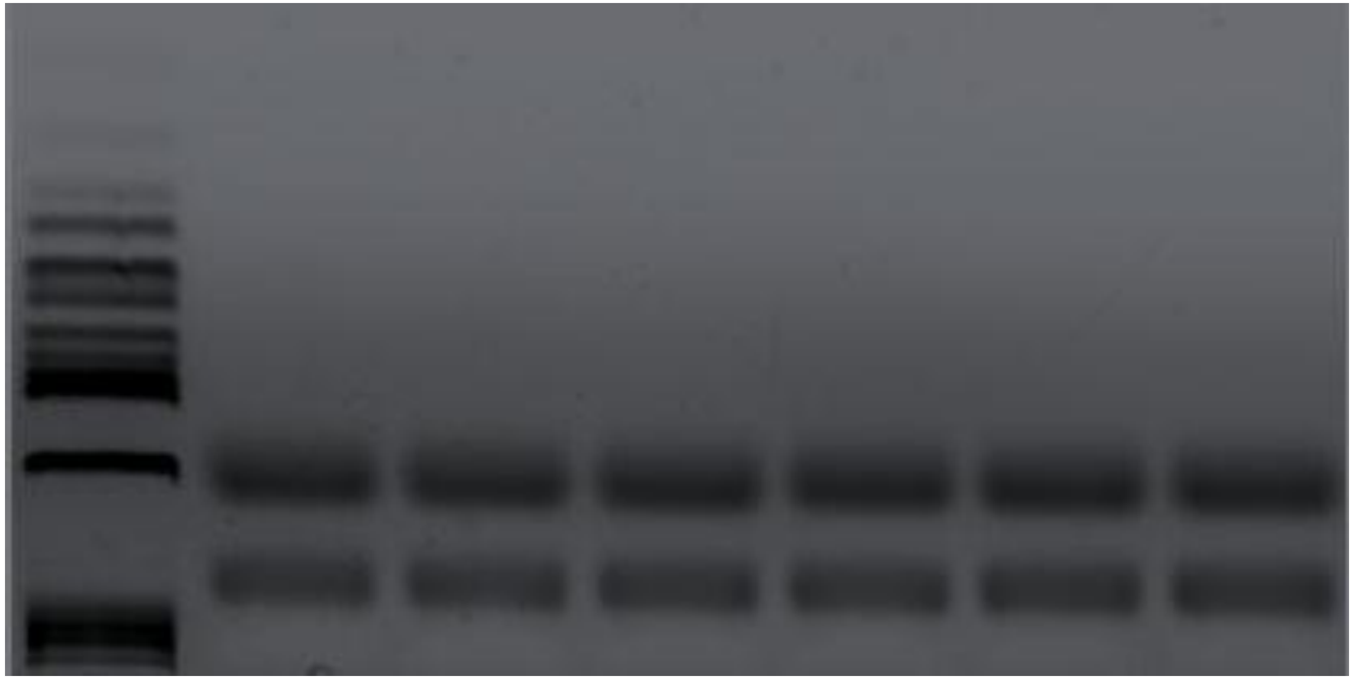

ERK-1
ERK-2

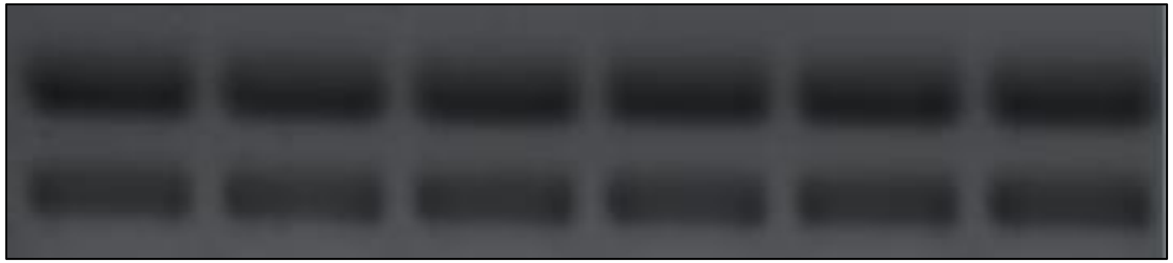

44 KDs
42 KDs

ERK 1,2

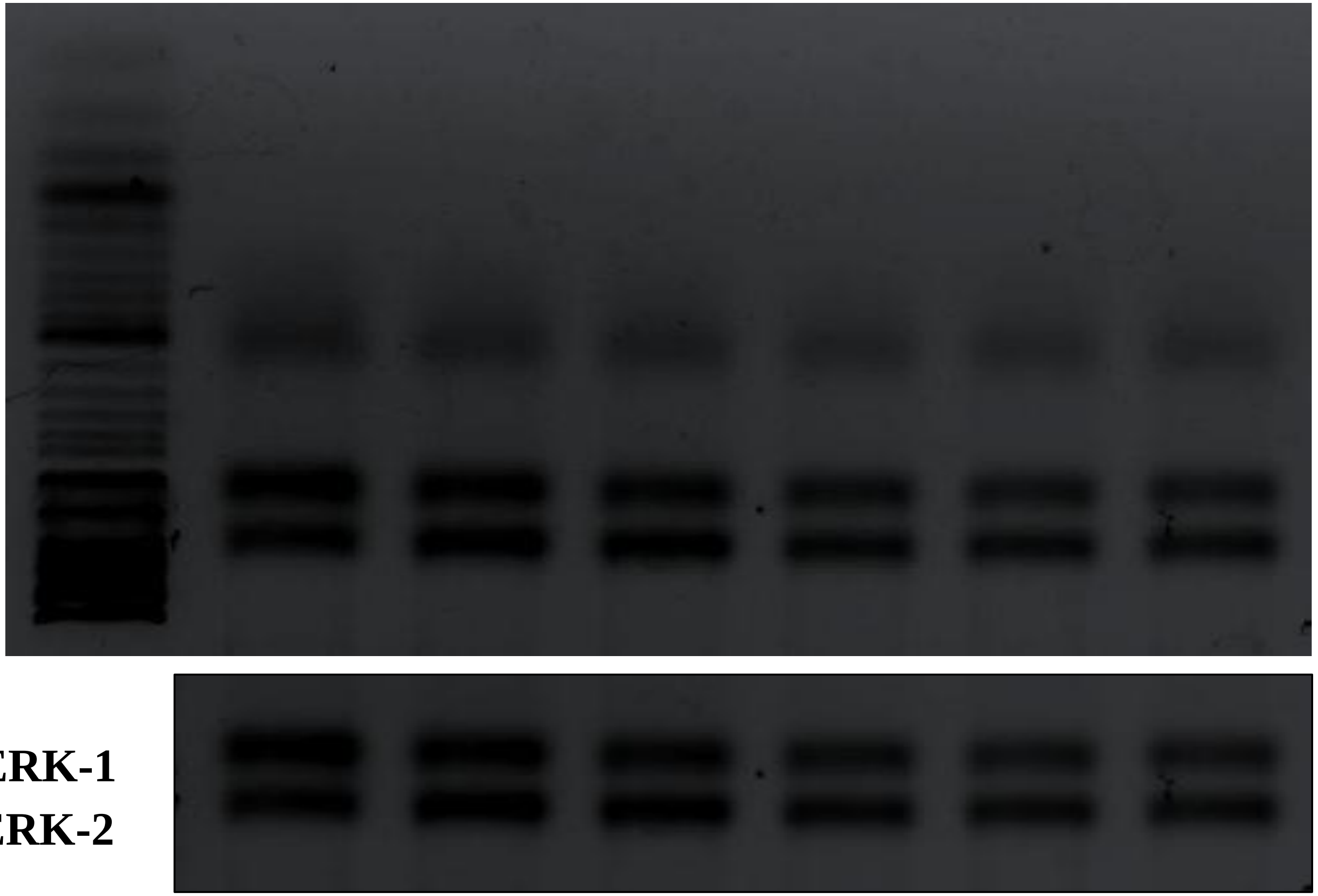

ERK-1 44 KDs

ERK-2 42 KDs

Nrf2

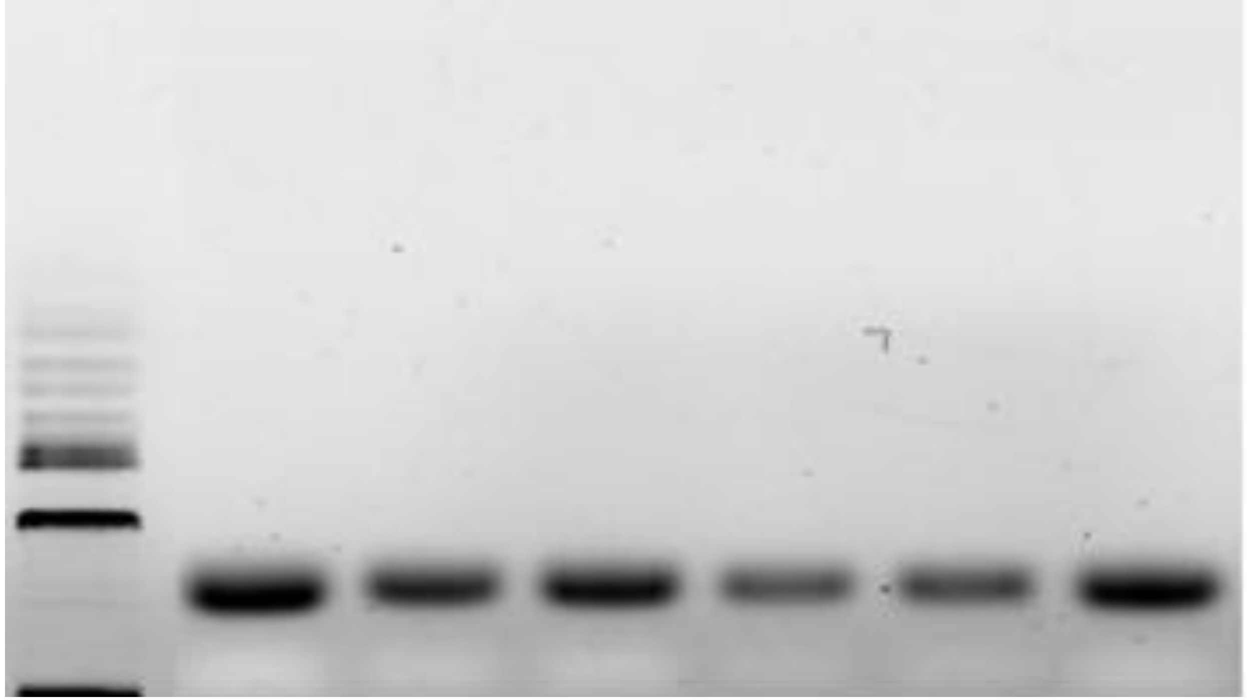

Nrf2

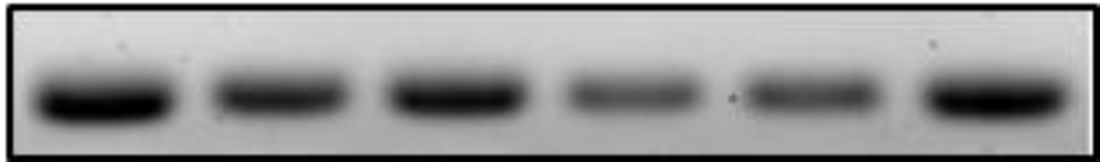

57 KDs

Nrf2

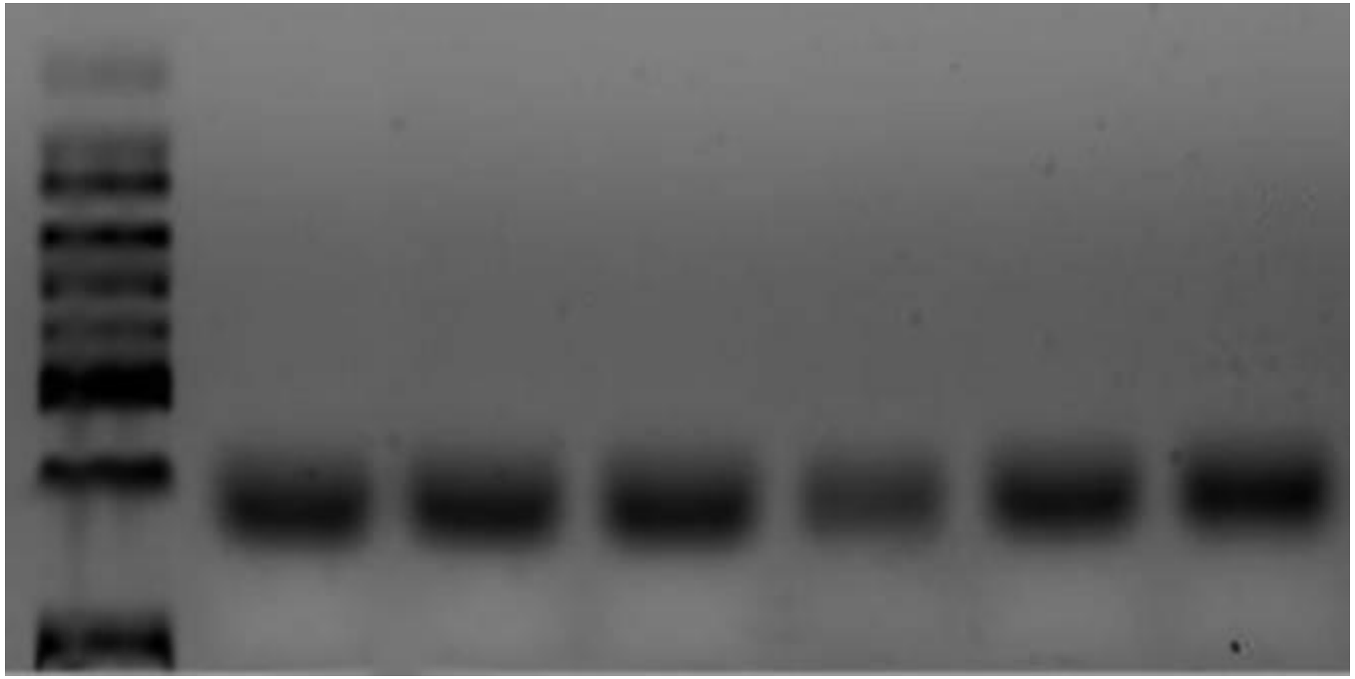

Nrf2

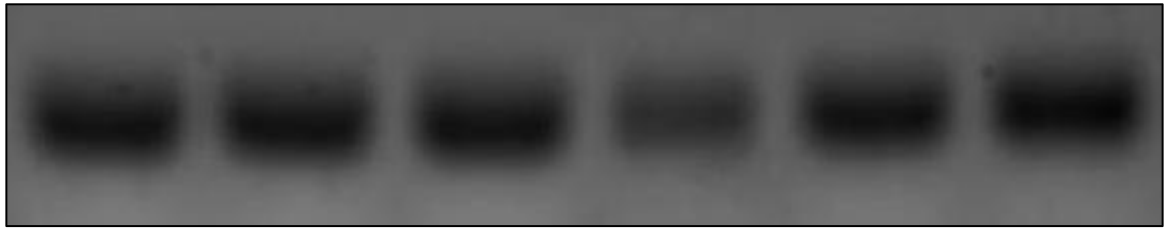

57 KDs

<u>**Nrf2**</u>

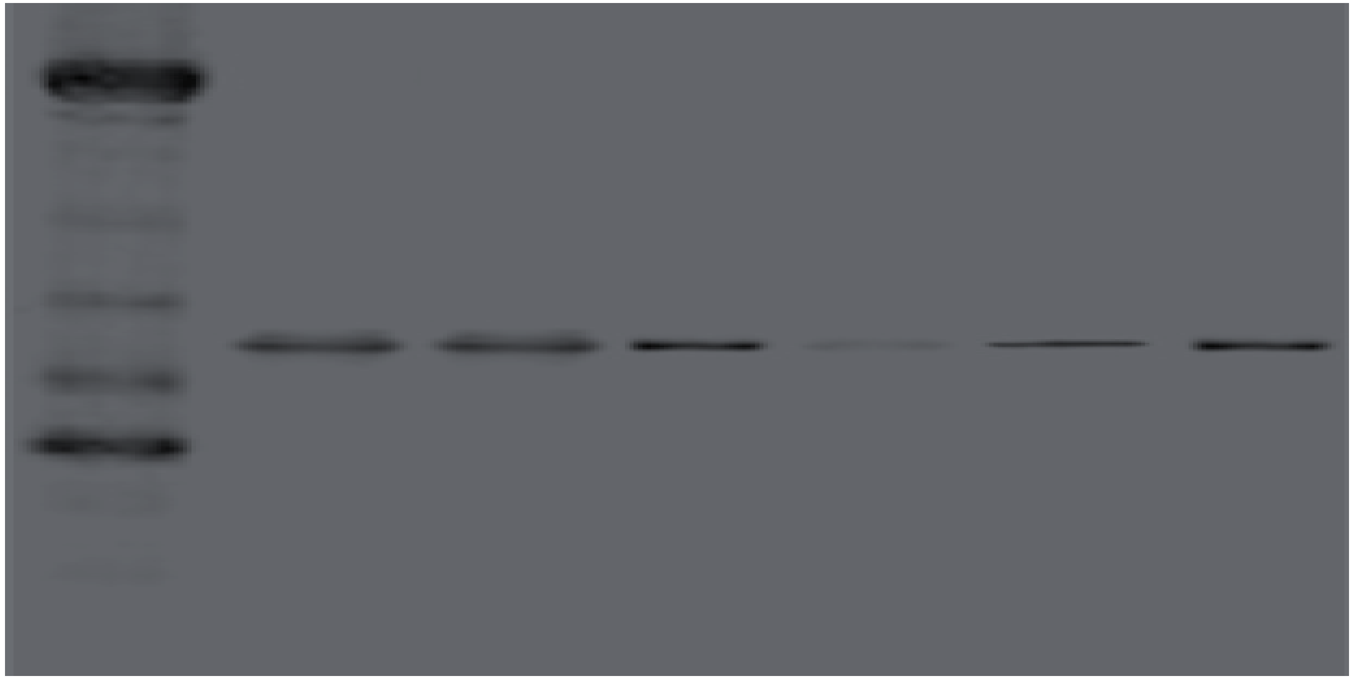

Nrf2

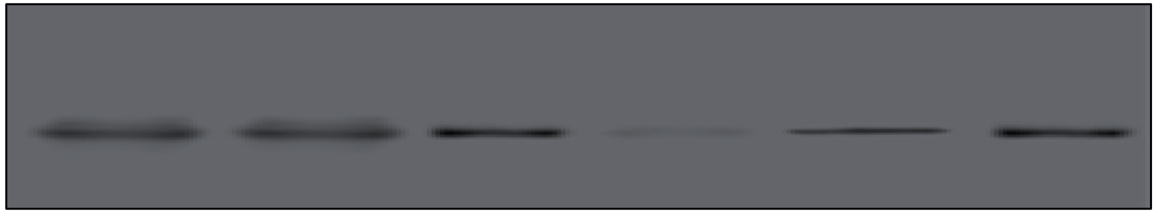

57 KDs

B-actin

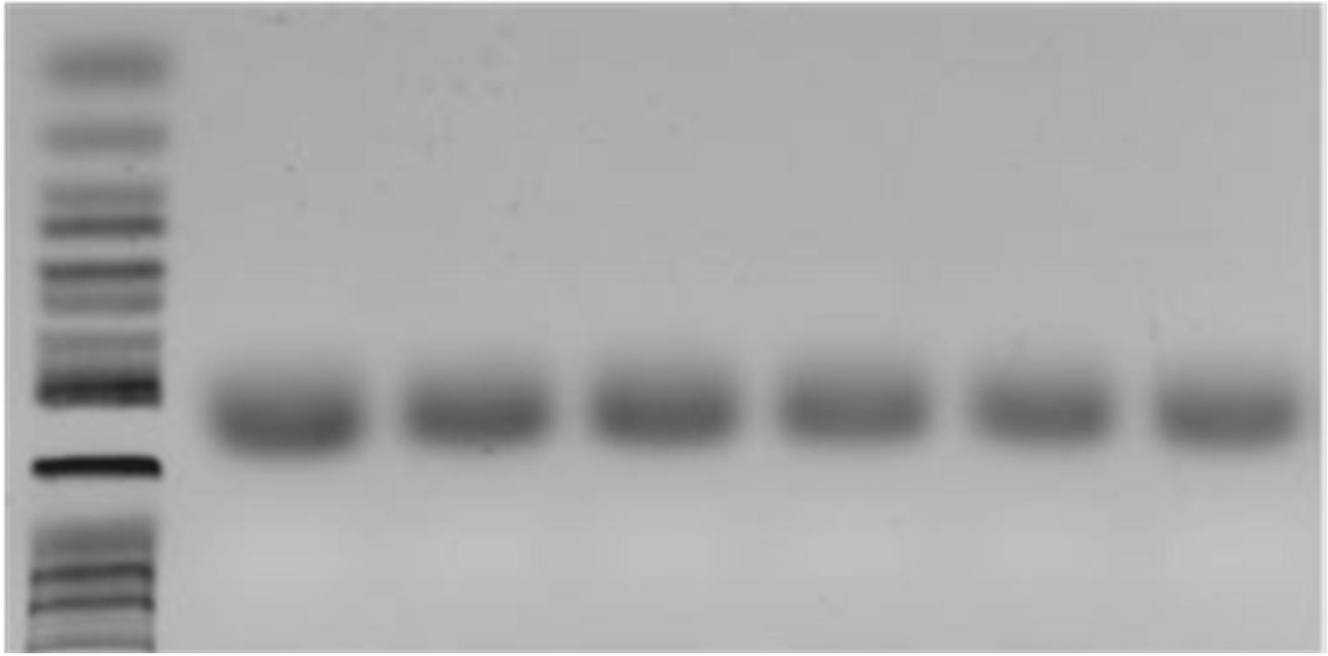

B-actin 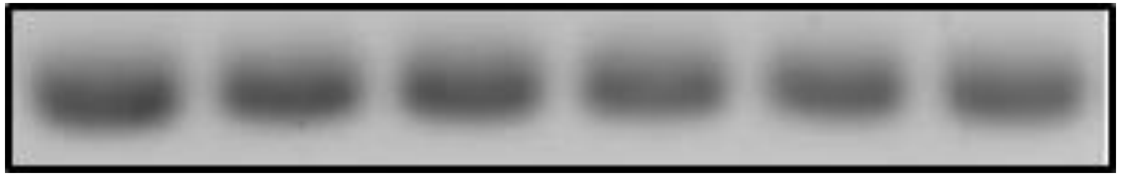 43 KDs

B-actin

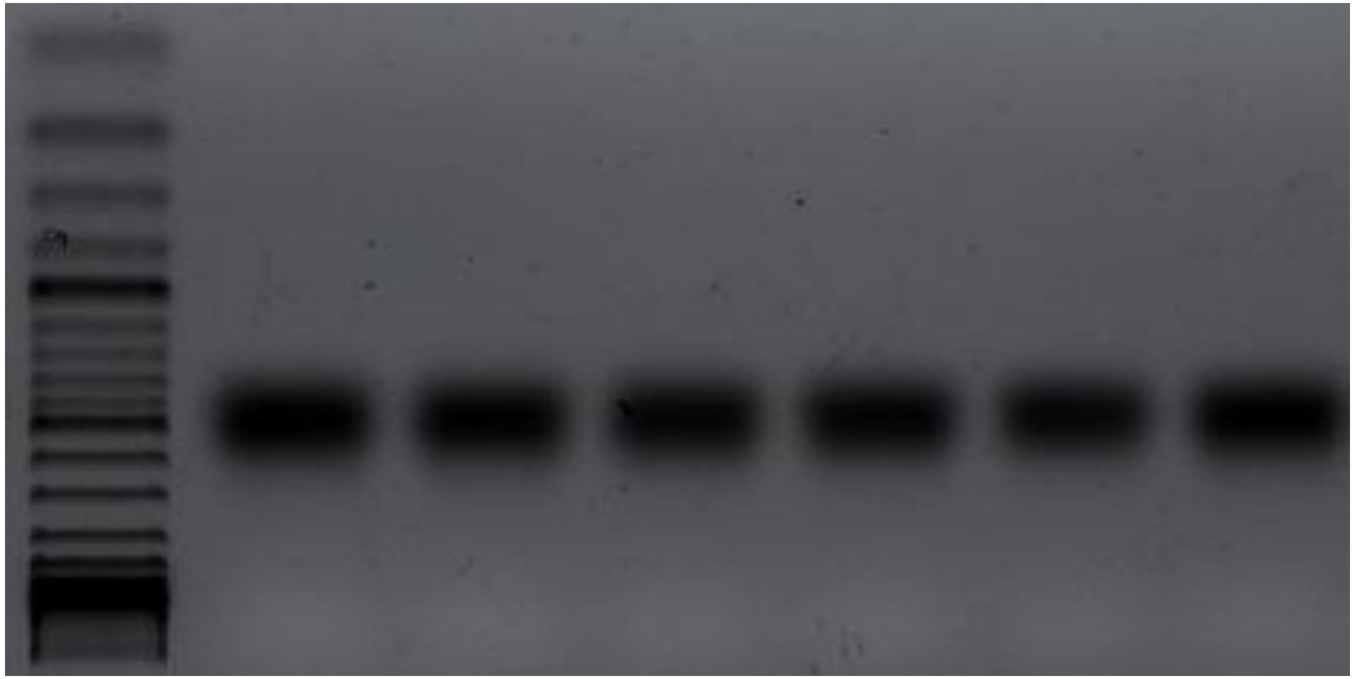

B-actin 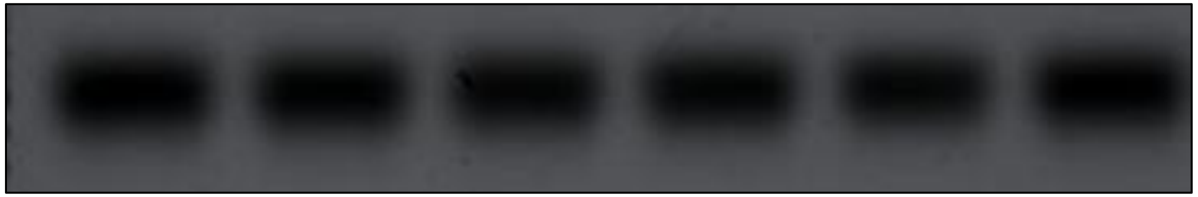 43 KDs

B-actin

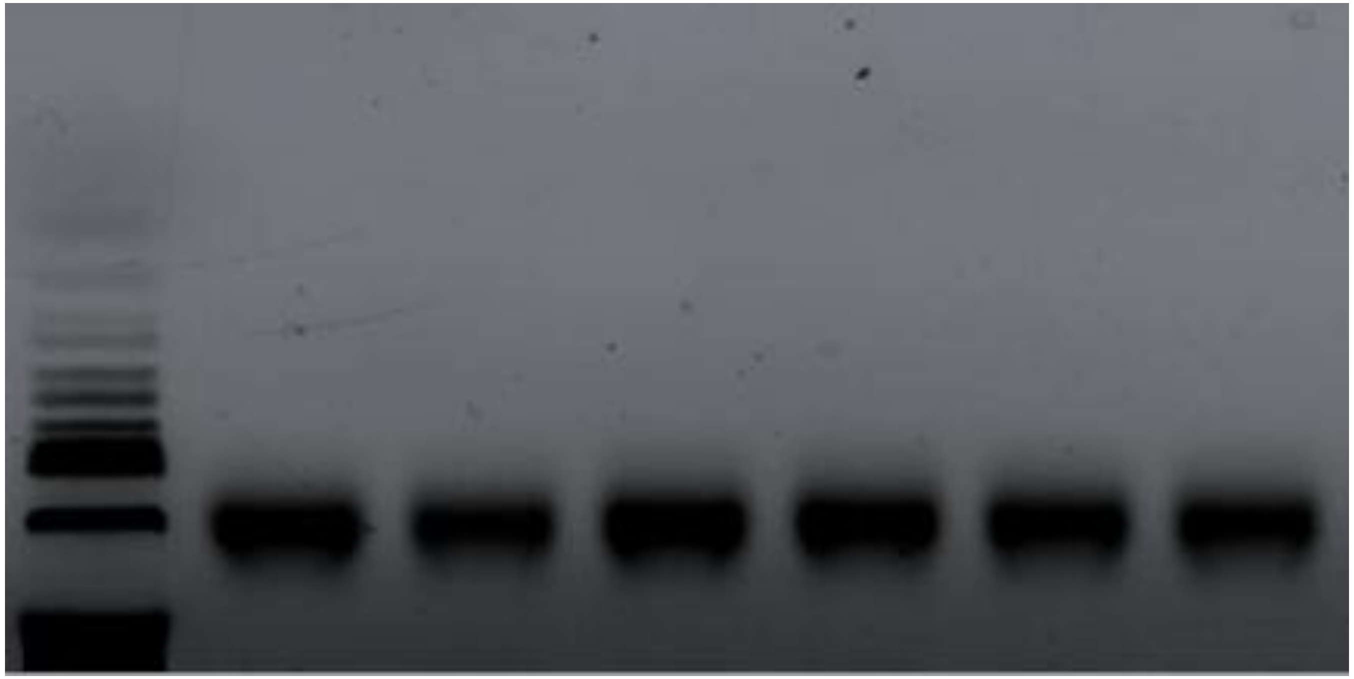

B-actin

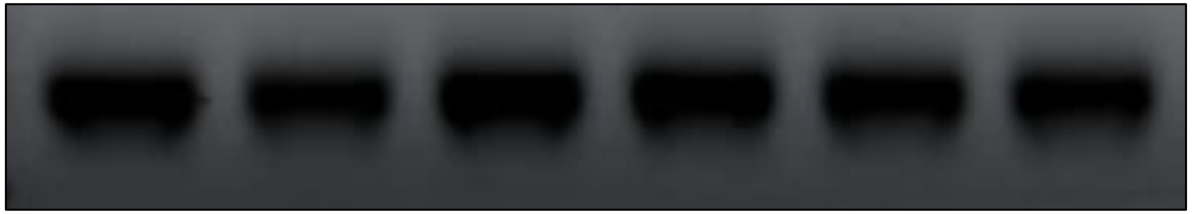

43 KDs
